# Clinical Characteristics and Prognostic Analysis of Hospitalized Infants with *Chlamydia trachomatis* Pneumonia

**DOI:** 10.3390/children13101416

**Published:** 2026-10-15

**Authors:** Ming Wei, Liwei Gao, Baoping Xu, Ju Yin, Qiang Qin, Anxia Jiao, Qi Gao, Xirong Wu, Siyuan Guo

**Affiliations:** 1 Department of Respiratory Disease, Beijing Children’s Hospital, Capital Medical University, National Center for Children’s Health, Beijing 100045, China; wming0102@163.com (M.W.); xubaoping@bch.com.cn (B.X.); yinju@bch.com.cn (J.Y.); qin-qiang@126.com (Q.Q.); gaoqibch@163.com (Q.G.); wuxirong2001@163.com (X.W.); gsygwx@163.com (S.G.); 2 China National Clinical Research Center of Respiratory Diseases, Beijing 100045, China; jiaoanxia@bch.com.cn; 3 Department of Interventional Pulmonology, Beijing Children’s Hospital, Capital Medical University, National Center for Children’s Health, Beijing 100045, China

**Keywords:** *Chlamydia trachomatis*, pneumonia, infant, clinical characteristics, imaging, prognosis

## Abstract

**Highlights:**

**What are the main findings?**

• Atypical chest imaging manifestations (mass-like or nodular opacities and diffuse miliary or granular shadows) are identified in a substantial proportion of infants with *Chlamydia trachomatis* pneumonia, expanding the recognized radiographic spectrum beyond classical interstitial infiltrates.
• Tachypnea and retractions are important early clinical indicators of severe disease, with retractions showing a statistically significant association with the need for respiratory support.

**What are the implications of the main findings?**

• Recognition of atypical radiographic patterns is critical to avoid misdiagnosis as invasive fungal infections, miliary tuberculosis, or neoplastic processes, thereby preventing unnecessary interventions and delays in appropriate antimicrobial therapy.
• Early identification of tachypnea and retractions can help clinicians stratify disease severity and guide timely respiratory support decisions in affected infants.

**Abstract:**

**Background/Objectives**: *Chlamydia trachomatis* (*C. trachomatis*) is an important cause of community-acquired pneumonia in early infancy; however, its subtle and nonspecific clinical features often result in underdiagnosis and delayed treatment. Evidence regarding clinical predictors of severe disease and distinctive imaging manifestations remains limited. This study aimed to characterize the clinical and imaging features of hospitalized infantile *C. trachomatis* pneumonia and to identify potential indicators associated with disease severity. **Methods**: This retrospective study enrolled infants with confirmed *C. trachomatis* pneumonia hospitalized at Beijing Children’s Hospital from January 2016 to December 2025. Data on demographics, clinical presentations, laboratory findings, chest imaging, treatments, and outcomes were systematically collected. Exploratory subgroup analyses were performed to compare severe and non-severe cases and identify severity-associated factors. **Results**: A total of 30 infants (16 boys, 14 girls) were included, with a median age of 34.0 days (IQR, 23.5–39.8 days) at symptom onset. Cough was nearly universal (96.7%), while fever, tachypnea, and wheezing were observed in 43.3%, 43.3%, and 40.0%, respectively. Pulmonary crackles were present in 86.7%, and eosinophilia in 40.0%. Cytomegalovirus co-infection occurred in 36.7%. Chest CT revealed patchy opacities (100%), interstitial changes (86.7%), mass-like/nodular opacities (63.3%), and diffuse miliary/granular opacities (13.3%). Among 14 patients undergoing bronchoscopy, 50.0% had thick airway secretions. Tachypnea and retractions emerged as important clinical indicators of severe disease. All patients recovered with macrolide therapy, and no deaths occurred. **Conclusions**: Infantile *C. trachomatis* pneumonia typically presents with absent or low-to-moderate fever, paroxysmal cough, and occasional wheezing. Tachypnea and retractions may serve as early indicators of severe disease. Atypical chest imaging patterns, including mass-like and miliary opacities, provide valuable diagnostic clues. Once the diagnosis is established, prompt initiation of macrolide therapy is recommended, and the overall prognosis is favorable.

## 1. Introduction

*Chlamydia trachomatis* (*C. trachomatis*) is a Gram-negative, obligate intracellular bacterium that relies entirely on host cells for energy production and replication [1,2,3]. It is among the most prevalent bacterial sexually transmitted pathogens worldwide [4,5,6,7]. In pregnant women, cervical *C. trachomatis* infection is frequently asymptomatic but is associated with a substantial risk of vertical transmission [8,9]. Among infants born vaginally to infected mothers, approximately 50–70% acquire the infection, of whom 30–50% develop conjunctivitis and up to 10–20% subsequently develop pneumonia [5,10]. Transmission occurs predominantly through intrapartum exposure to infected genital secretions. Nevertheless, infection has also been reported in approximately 20% of infants delivered by cesarean section, suggesting alternative mechanisms such as ascending intrauterine infection or perinatal exposure to contaminated amniotic fluid [1,11]. Maternal *C. trachomatis* infection is usually diagnosed by nucleic acid amplification testing of cervical or vaginal swabs and can be effectively treated with macrolides such as azithromycin [1,3,4,7]. Antenatal screening and treatment of infected mothers are therefore key measures to prevent neonatal *C. trachomatis* infection.

*C. trachomatis* pneumonia is an important cause of lower respiratory tract infection during early infancy [11]. In an etiological study of 1408 infants under 6 months of age with pneumonia in Shenzhen, China, approximately 7.2% were infected with *C. trachomatis* [12]. Disease onset typically occurs between 4 and 12 weeks of age and is classically characterized by absent or low-grade fever, pertussis-like paroxysmal cough, tachypnea, and occasional wheezing. However, the clinical manifestations are often subtle and nonspecific, which may contribute to underdiagnosis or misdiagnosis as viral bronchiolitis or other forms of community-acquired pneumonia. Furthermore, a subset of affected infants may develop severe disease requiring intensive care and respiratory support, particularly in the presence of underlying comorbidities or mixed infections.

Despite its clinical relevance, detailed evidence regarding the clinical spectrum, imaging characteristics, and prognostic factors of infantile *C. trachomatis* pneumonia remains limited. In particular, potential indicators of severe disease and distinctive imaging manifestations, including mass-like opacities and diffuse miliary patterns, have not been comprehensively characterized. Therefore, this study aimed to systematically describe the clinical manifestations, laboratory findings, chest imaging features, treatment, and prognosis of infantile *C. trachomatis* pneumonia and to explore potential early indicators of severe disease requiring respiratory support. By raising clinical awareness of this underrecognized entity, we hope to facilitate earlier diagnosis, avoid unnecessary investigations, and improve management strategies for affected infants.

## 2. Materials and Methods

### 2.1. Study Subjects

We retrospectively included pediatric patients diagnosed with *C. trachomatis* pneumonia who were hospitalized at Beijing Children’s Hospital, Capital Medical University, between January 2016 and December 2025. Eligibility criteria were as follows: (1) clinical and radiographic findings consistent with pneumonia according to the 2024 Chinese Guidelines for Management of Community-Acquired Pneumonia in Children [13]; community-acquired pneumonia was defined as pneumonia acquired outside the hospital in a previously healthy child, and diagnosis required at least two of the following: (a) clinical symptoms (cough, sputum production, worsening respiratory symptoms, fever, tachypnea, grunting, or cyanosis); (b) signs (fixed pulmonary crackles and/or consolidation, with possible nasal flaring or chest wall retractions); (c) chest imaging (infiltrates, lobar or segmental consolidation, ground-glass opacities, or interstitial changes, with or without pleural effusion). (2) Confirmation of *C. trachomatis* infection by at least one etiological method, including a positive nucleic acid amplification test of a respiratory specimen, detection of *C. trachomatis*-specific sequences by metagenomic next-generation sequencing (mNGS) of bronchoalveolar lavage fluid (BALF), positive serum anti-*C. trachomatis* IgM, or a ≥4-fold increase in IgG titers between paired serum samples. Of note, not all patients underwent the same panel of etiological tests; confirmation could be achieved by any one of the methods listed above. (3) Availability of complete medical records, including medical history, physical examination findings, laboratory data, and chest imaging. The study was approved by the institutional ethics committee, which waived the requirement for informed consent (approval number: 2026-Y-060-D).

### 2.2. Data Collection

Data were extracted from electronic medical records and included demographic characteristics and perinatal characteristics (sex, age, mode of delivery, birth weight, gestational age, and maternal history of vaginitis), age at symptom onset, duration of illness before admission, and length of hospital stay; clinical manifestations (fever, cough, tachypnea, wheezing, apnea, pertussis-like paroxysmal cough, frothing at the mouth, feeding-associated regurgitation or choking, and ocular discharge); and physical examination findings (pulmonary crackles, wheezes, retractions, and cyanosis). Laboratory variables included white blood cell (WBC) and eosinophil counts, hemoglobin (Hb), C-reactive protein (CRP), procalcitonin (PCT), erythrocyte sedimentation rate (ESR), arterial partial pressure of carbon dioxide (PaCO_2_), liver enzymes (alanine aminotransferase [ALT] and aspartate aminotransferase [AST]), myocardial enzymes (creatine kinase [CK], creatine kinase-MB [CK-MB], and lactate dehydrogenase [LDH]), D-dimer, and immunoglobulins (IgG and IgM). Etiologic findings and co-infections, chest imaging and bronchoscopic findings, treatment details (antibiotic regimen and duration), and clinical outcomes were also recorded.

### 2.3. Definitions

Severe pneumonia was defined according to the 2024 Chinese Guidelines for Management of Community-Acquired Pneumonia in Children [13]. Patients were classified as having severe disease if any of the following criteria were present: poor general response, feeding refusal or dehydration, impaired consciousness, marked tachypnea (≥70 breaths/min in infants), cyanosis, dyspnea, pulse oximetric saturation ≤ 0.92, multilobar or extensive pulmonary infiltration involving ≥2/3 of the lung, pleural effusion, or extrapulmonary complications. Eosinophilia was defined as an absolute eosinophil count > 0.5 × 10^9^/L. Mixed infection was defined as concurrent detection of at least one additional viral or bacterial respiratory pathogen in addition to *C. trachomatis*.

### 2.4. Statistical Analysis

Statistical analyses were performed using R version 4.6.1. Continuous variables were compared using the Wilcoxon rank-sum test, whereas categorical variables were compared using Fisher’s exact test. Univariate logistic regression analysis was performed to identify potential risk factors for severe disease, with results reported as odds ratios (OR) with 95% confidence intervals (CIs). Due to the limited sample size, multivariate logistic regression analysis was not performed. All tests were two-sided, and a *p* value < 0.05 was considered statistically significant.

## 3. Results

### 3.1. Baseline Characteristics

A total of 30 patients were included, of whom 16 (53.3%) were boys and 14 (46.7%) were girls. The median age at admission was 53.0 days (IQR, 43.5–62.0 days), and all infants were born at term. Twenty-eight patients (93.3%) were delivered vaginally, whereas 2 (6.7%) were delivered by cesarean section, including one emergency cesarean delivery following a failed trial of labor. The median birth weight was 3.5 kg (IQR, 3.1–3.7 kg). A maternal history of vaginitis or increased vaginal discharge during pregnancy was documented in 15 cases (50.0%). The median age at symptom onset was 34.0 days (IQR, 23.5–39.8 days), the median duration of illness before admission was 20.0 days (IQR, 10.3–29.0 days), and the median length of hospital stay was 12.0 days (IQR, 9.0–15.8 days). Baseline characteristics are summarized in Table 1.

### 3.2. Clinical Manifestations

**Figure 1:**
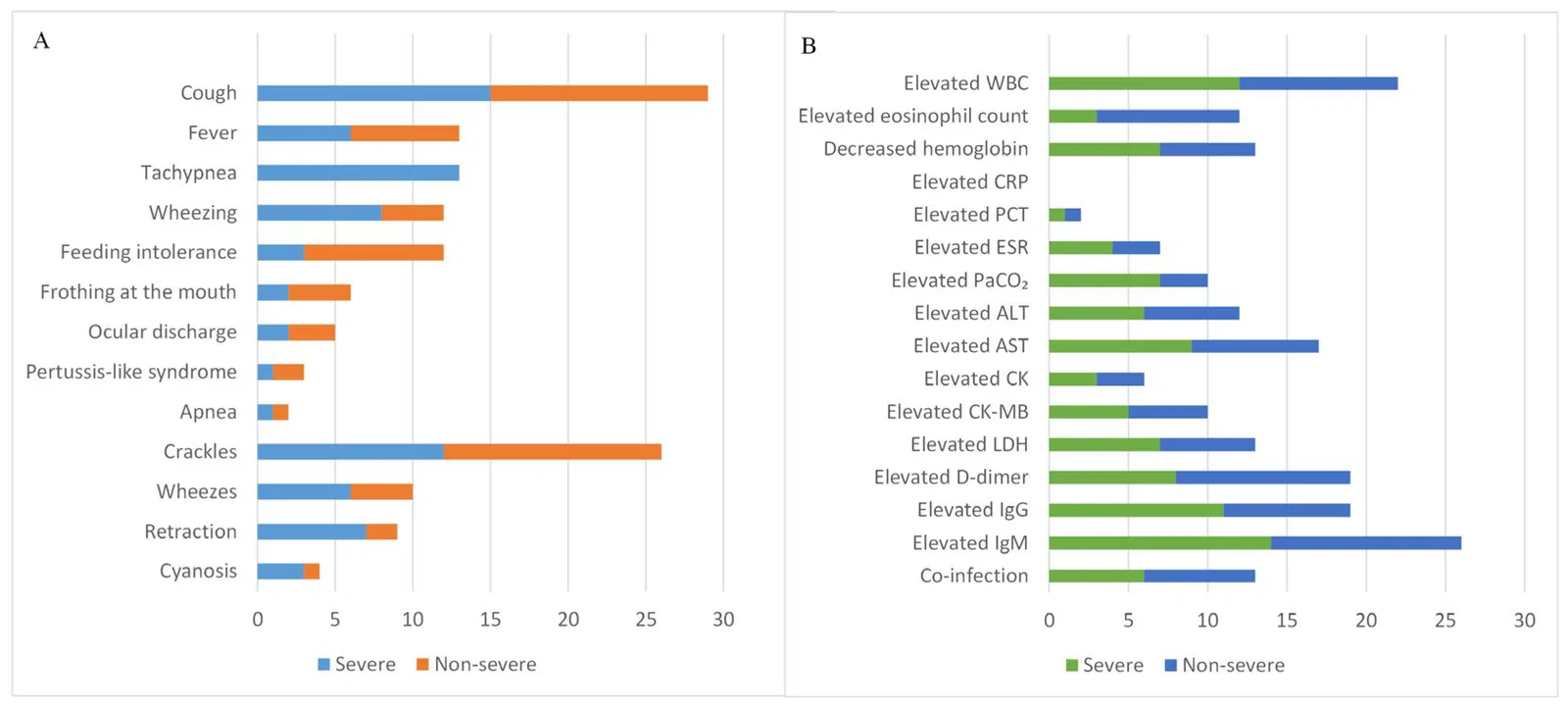
Distribution of clinical characteristics and laboratory findings in 30 infants. (**A**) Clinical symptoms and signs; (**B**) laboratory findings. Abbreviations: WBC, white blood cell; CRP, C-reactive protein; PCT, procalcitonin; ESR, erythrocyte sedimentation rate; PaCO_2_, arterial partial pressure of carbon dioxide; ALT, alanine aminotransferase; AST, aspartate aminotransferase; CK, creatine kinase; CKMB, creatine kinase-MB; LDH, lactate dehydrogenase; IgG, immunoglobulin G; IgM, immunoglobulin M.

Cough was the predominant symptom, occurring in 29 patients (96.7%), including pertussis-like paroxysmal cough in 3 (10.0%). Fever was documented in 13 patients (43.3%) and was generally low to moderate in severity (range, 37.4–38.5 °C). Tachypnea occurred in 13 patients (43.3%), wheezing in 12 (40.0%), and feeding-associated regurgitation or choking in 12 (40.0%). Additional manifestations included frothing at the mouth in 6 cases (20.0%), ocular discharge in 5 (16.7%), and apnea in 2 (6.7%). On physical examination, pulmonary crackles were detected in 26 patients (86.7%), wheezes in 10 (33.3%), retractions in 9 (30.0%), and cyanosis in 4 (13.3%). The distribution of clinical symptoms and physical examination findings is presented in Figure 1A.

### 3.3. Laboratory Findings

Elevated WBC counts were observed in 22 patients (73.3%), with a median WBC count of 11.83 × 10^9^/L (IQR, 9.43–14.29 × 10^9^/L; range, 7.12–33.74 × 10^9^/L). Eosinophilia was present in 12 patients (40.0%), with a median eosinophil count of 0.46 × 10^9^/L (IQR, 0.21–0.84 × 10^9^/L; range, 0–2.78 × 10^9^/L). Anemia was identified in 13 patients (43.3%), with a median Hb concentration of 103.5 g/L (IQR, 93.8–110.0 g/L; range, 76–152 g/L). Inflammatory markers were generally low: CRP levels were normal in all patients, whereas PCT was elevated in 2 of 22 patients tested (6.7%) and ESR in 7 of 23 (30.4%). Blood gas analysis demonstrated elevated PaCO_2_ in 10 of 28 patients tested (35.7%). Liver enzyme abnormalities were frequent, with elevated AST in 17 patients (56.7%) and ALT in 12 (40.0%). Elevations in myocardial enzymes included LDH in 13 patients (43.3%), CK-MB in 10 (33.3%), and CK in 6 (20.0%). D-dimer was elevated in 19 of 29 patients tested (65.5%). Immunoglobulin testing showed elevated IgM in 26 of 27 patients (96.3%) and elevated IgG in 19 of 27 tested (70.4%). Abnormal laboratory findings are summarized in Figure 1B.

### 3.4. Etiologic Findings

Elevated *C. trachomatis*-specific IgM antibodies were detected in 13 patients (43.3%). *C. trachomatis* nucleic acid was detected in throat swabs or BALF in 7 patients (23.3%), while mNGS of BALF detected *C. trachomatis* in an additional 7 patients (23.3%). Four patients tested positive by more than one diagnostic method. Co-infection with other pathogens was identified in 13 patients (43.3%). *Cytomegalovirus* (CMV) was the most frequent co-pathogen, detected in 11 cases (36.7%). Other co-infecting pathogens included respiratory syncytial virus in 3 cases (10.0%), oral *Candida* species in 2 (6.7%), and parainfluenza virus, coronavirus, *Mycoplasma pneumoniae*, and *Pneumocystis jirovecii* in 1 case each, among others. *Bordetella pertussis* testing was performed in all patients, and all results were negative.

### 3.5. Chest Imaging Findings

**Figure 2:**
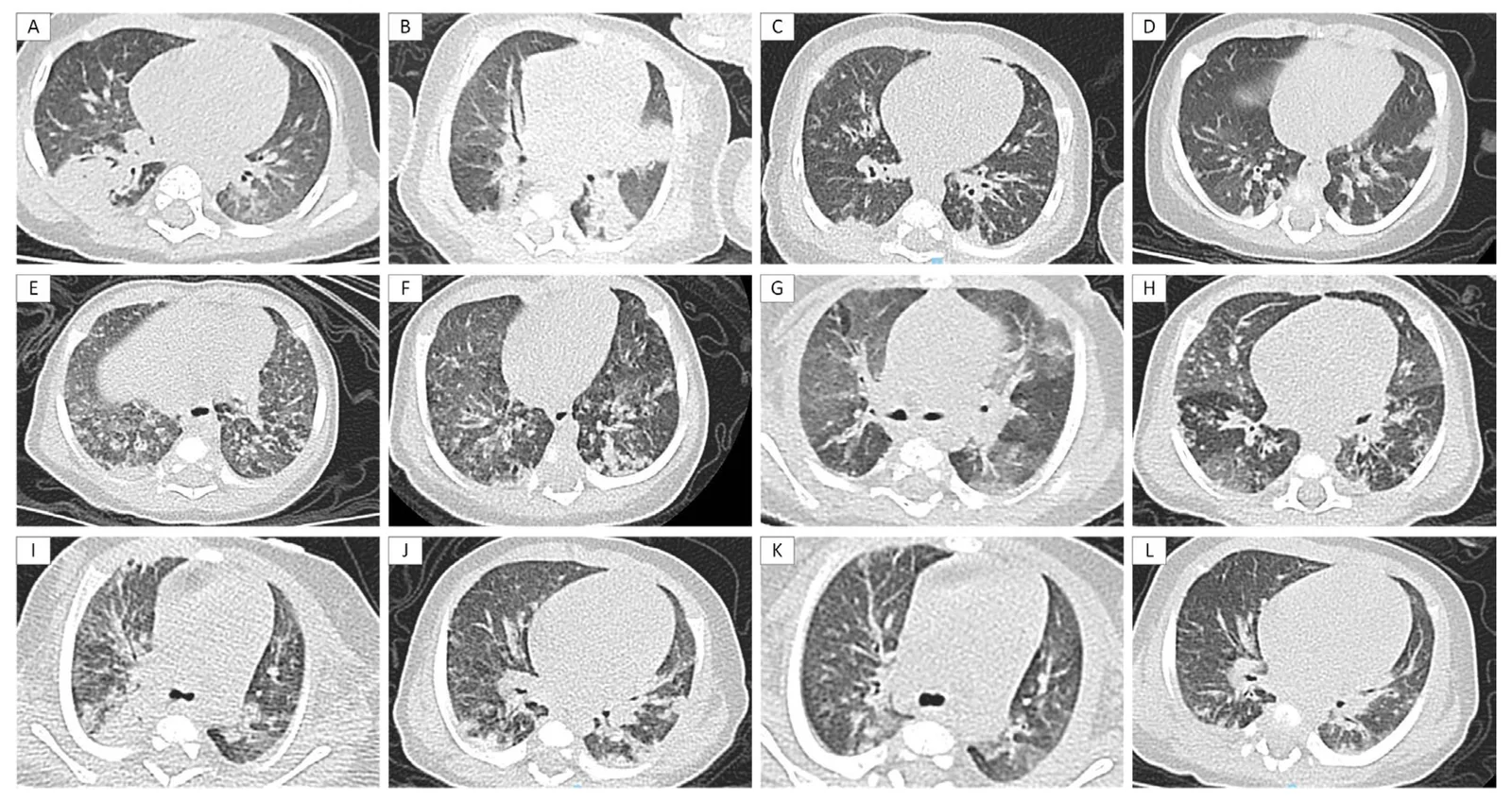
Representative HRCT imaging features in patients with *C. trachomatis* pneumonia. (**A**,**B**) Mass-like opacities with well-defined margins in patient 1 (**A**) and patient 22 (**B**). (**C**,**D**) Patchy opacities with ill-defined margins and subpleural nodules in patient 21 (**C**) and patient 14 (**D**). (**E**,**F**) Diffuse miliary/granular shadows in patient 5 (**E**) and patient 11 (**F**). (**G**,**H**) Regional inhomogeneity of lung lucency in patient 4 (**G**) and patient 25 (**H**). (**I**,**J**) Pre-treatment chest HRCT of a representative patient 6 showing a mass-like opacity in the right upper lobe. (**K**,**L**) Post-treatment chest CT of patient 6 showing significant lesion resolution.

High-resolution computed tomography (HRCT) demonstrated patchy opacities in all 30 patients (100%). Other abnormalities included interstitial and parenchymal infiltrates (86.7%), regional inhomogeneity of lung lucency (76.7%), mass-like or nodular opacities (63.3%), diffuse miliary or granular opacities (13.3%), pulmonary emphysema (6.7%), and bronchiectasis-like changes (3.3%). Small nodular opacities and air bronchograms were also observed in some patients. Bilateral pulmonary involvement was present in all 30 patients (100%). Representative HRCT findings are shown in Figure 2. Notably, patients 1, 5, and 4 were co-infected with CMV, while patients 22, 11, and 25 had no co-infection, yet both groups exhibited similar imaging patterns, suggesting that these HRCT features are primarily attributable to *C. trachomatis* infection rather than co-infection.

**Figure 3:**
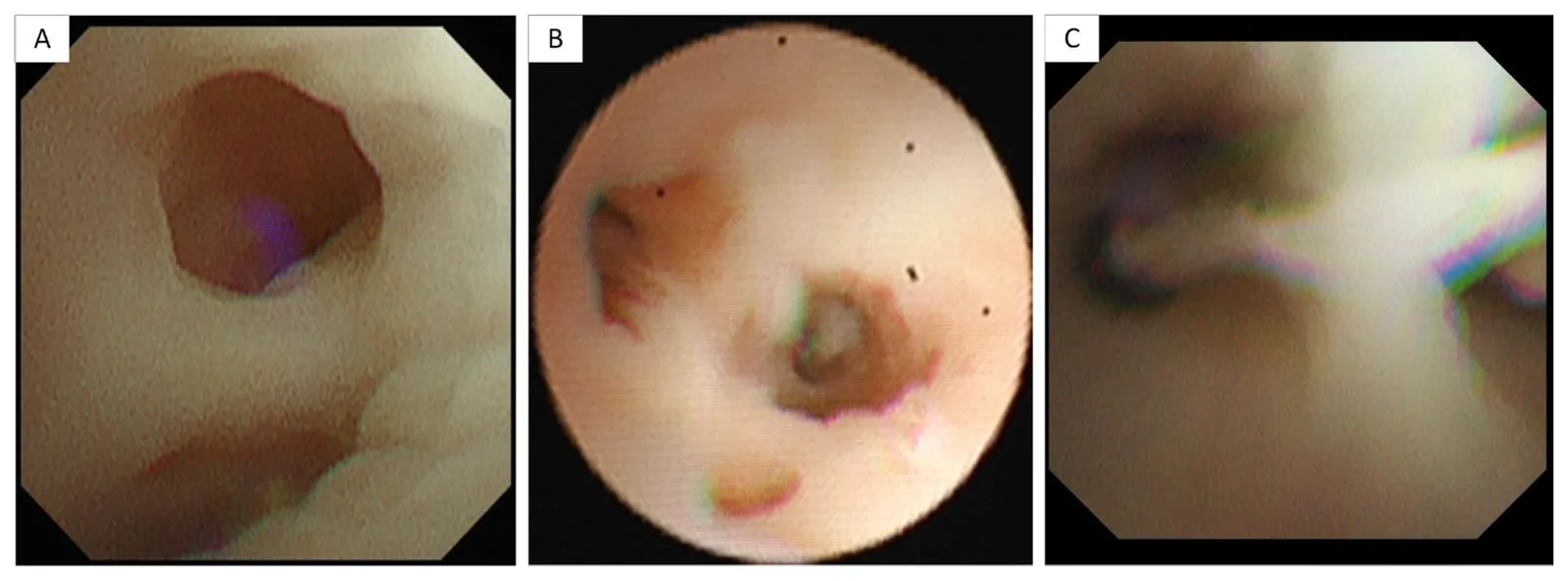
Bronchoscopy revealed increased viscous secretions in the trachea and bronchi of patient 1 (**A**), patient 2 (**B**) and patient 30 (**C**).

Bronchoscopy was performed in 14 patients. Thick mucoid secretions were observed in 7 patients (50.0%), of whom 2 also exhibited marked mucosal edema (Figure 3).

### 3.6. Severe vs. Non-Severe Comparison

Tachypnea was significantly more frequent in patients with severe disease than in those with non-severe disease (92.9% vs. 0.0%, *p* < 0.001). Univariate logistic regression showed that retractions showed a statistically significant association with severe disease (OR 7.000, 95% CI 1.296–56.189, *p* = 0.036), whereas wheezing and feeding-associated regurgitation or choking demonstrated borderline associations (*p* < 0.1). Detailed results are presented in Table 2 and Supplementary Table S1.

Given the limited sample size (n = 30), multivariable logistic regression was not performed to minimize the risk of model overfitting. Accordingly, the univariate findings should be considered exploratory and require validation in larger cohorts.

### 3.7. Treatment and Outcomes

Most patients (24/30, 80.0%) received erythromycin for 2 weeks; treatment was extended to 3 weeks in patients with persistent fever, prominent cough or wheezing, or inadequate radiologic resolution. Among patients with severe disease, 6 required continuous positive airway pressure-assisted respiratory support, including 2 who subsequently underwent endotracheal intubation. Of the 11 children with CMV co-infection, 7 received ganciclovir, whereas the remaining 4 were considered to have asymptomatic infection and therefore did not receive antiviral therapy. One child with Pneumocystis jirovecii co-infection was treated with trimethoprim–sulfamethoxazole. All patients became afebrile and experienced marked improvement in cough. Follow-up imaging demonstrated substantial resolution of pulmonary infiltrates (Figure 2). Long-term follow-up data were available for 26 of the 30 infants, with some followed for more than 10 years. All remained in good general condition, with no recurrence of cough or wheezing.

## 4. Discussion

Infantile *C. trachomatis* pneumonia is an underrecognized but clinically important cause of lower respiratory tract infection in early infancy. Its nonspecific presentation and atypical imaging patterns frequently lead to misdiagnosis and delayed treatment. The present study aimed to characterize the clinical manifestations, laboratory findings, chest imaging features, treatment, and outcomes of infantile *C. trachomatis* pneumonia, with particular attention to potential early indicators of severe disease requiring respiratory support. Improved recognition of this entity may facilitate earlier diagnosis, reduce unnecessary investigations for fungal or tuberculous infection, and promote timely macrolide therapy, thereby potentially improving both short-term outcomes and long-term respiratory health.

Infantile *C. trachomatis* pneumonia predominantly affected full-term infants within the first 3 months of life and generally had an insidious onset. Cough was the most common presenting symptom, pulmonary crackles were frequently detected on auscultation, and eosinophilia emerged as a potentially useful diagnostic clue. HRCT most commonly demonstrated bilateral patchy opacities and interstitial infiltrates; however, atypical patterns, including mass-like or nodular opacities and diffuse miliary shadows, were also observed in a substantial proportion of patients and may mimic fungal or tuberculous infection. Severe pneumonia occurred in 46.7% of the cohort (14/30), and clinical signs of respiratory distress were associated with greater disease severity. Despite this relatively high proportion of severe cases, the overall prognosis was favorable, with no deaths following standard macrolide therapy.

The median age at symptom onset was 34 days, consistent with the well-recognized 4-to-12-week window for infantile *C. trachomatis* pneumonia. In a Brazilian cohort of 151 infants, *C. trachomatis* was detected exclusively in infants younger than 5 months, with the highest prevalence during the first 2 months of life [14]. Similarly, a retrospective study from Xiamen, China, reported that 88.0% of 117 cases occurred in infants younger than 3 months, with severe pneumonia developing in 28.2% [15]. Vertical transmission remains the predominant route of infection. In the present cohort, 93.3% of infants were delivered vaginally, and 50% had a maternal history of vaginitis or increased vaginal discharge during pregnancy, reinforcing the potential importance of antenatal *C. trachomatis* screening for reducing mother-to-child transmission [8]. Notably, infection also occurred in two infants delivered by cesarean section, which may reflect ascending intrauterine infection or exposure associated with premature rupture of membranes and is consistent with reports that approximately 20% of infants delivered by cesarean section may nevertheless acquire infection [11,12]. These observations highlight the need for strengthened antenatal screening and timely maternal treatment. Future multicenter prospective studies, in collaboration with obstetric and public health departments, are needed to define regional prevalence, determine the optimal screening strategy, and clarify the role of maternal treatment in preventing neonatal infection.

The clinical profile observed in this cohort was broadly consistent with previous descriptions of infantile *C. trachomatis* pneumonia. Fever occurred in 43.3% of patients and was generally low-grade, whereas cough was present in 96.7% and pulmonary crackles were detected in 86.7%. Nevertheless, these manifestations are nonspecific and can readily be mistaken for viral bronchiolitis. Wheezing was observed in 40.0% of patients and warrants particular attention. Although earlier studies characterized wheezing as relatively uncommon in *C. trachomatis* pneumonia, more recent evidence suggests that it may occur more frequently than previously recognized, particularly among infants with an atopic predisposition [12,16]. Laboratory findings also provided potentially informative diagnostic clues. Eosinophilia was present in 40% of patients, while IgM and IgG levels were elevated in 26 cases (96.3%) and 19 cases (70.4%), respectively, consistent with previous reports [17]. Eosinophilia may reflect a delayed allergic response and may therefore aid differential diagnosis [11,18], whereas elevated IgM and IgG levels are indicative of a humoral immune response to *C. trachomatis* infection. Collectively, careful assessment of the clinical history, birth history, physical findings, and eosinophil count may facilitate earlier recognition of this condition.

The HRCT findings in this cohort further expand the recognized radiologic spectrum of infantile *C. trachomatis* pneumonia. Bilateral pulmonary involvement was present in all patients, with patchy opacities (100%) and interstitial or parenchymal infiltrates (86.7%) representing the predominant patterns. Of particular note, mass-like or nodular opacities were identified in 63.3% of patients and diffuse miliary shadows in 13.3%. These atypical findings are clinically important because, in young infants, they may initially raise concern for fungal infection, tuberculosis, or metastatic disease, potentially resulting in diagnostic delay or unnecessary investigations. Previous studies have focused primarily on interstitial infiltrates in *C. trachomatis* pneumonia, whereas mass-like and miliary patterns have been reported less frequently. Potential mechanisms include localized accumulation of inflammatory exudates, peribronchiolar granuloma formation, or atelectasis secondary to mucus plugging [18]. Accordingly, *C. trachomatis* pneumonia should be considered in the differential diagnosis when these atypical HRCT patterns are encountered in young infants, particularly to avoid unwarranted antifungal or antituberculous treatment.

Severe pneumonia occurred in 46.7% of patients (14/30). Tachypnea and retractions emerged as important clinical indicators of severe disease. This association is physiologically plausible because recruitment of accessory respiratory muscles reflects increased ventilatory effort and may signal progression toward respiratory decompensation. Previous studies have suggested that congenital heart disease, mixed infection, peripheral white blood cell counts > 15 × 10^9^/L, and elevated PaCO_2_ may be associated with severe infantile *C. trachomatis* pneumonia [15]. None of these factors reached statistical significance in the present cohort, potentially because of the limited sample size. Notably, among the 11 exclusively breastfed infants, only 4 developed severe pneumonia. Although this difference did not reach statistical significance, possibly due to the limited sample size, it is biologically plausible that breastfeeding may confer protection against severe *C. trachomatis* pneumonia through maternal secretory IgA and other immune factors. Mixed infections were identified in 43.3% of patients, with CMV representing the most frequent co-pathogen (36.7%), consistent with previous reports [14]. However, mixed infection was not significantly associated with severe disease in the subgroup analysis. This finding suggests that CMV co-detection in some infants may represent asymptomatic carriage rather than a major determinant of clinical deterioration.

Early recognition and appropriate treatment are important not only for controlling acute symptoms but also for potentially reducing long-term pulmonary sequelae [8]. Untreated *C. trachomatis* pneumonia may persist for weeks to months and has been associated with chronic cough, feeding difficulties, and poor weight gain. Persistent abnormalities in pulmonary function testing have also been reported during follow-up in some patients [19,20]. These observations support timely initiation of appropriate antimicrobial therapy to promote both short-term recovery and long-term respiratory health.

All patients in the present cohort received erythromycin for 2–3 weeks and demonstrated substantial clinical and radiologic improvement, supporting the effectiveness of macrolide therapy for infantile *C. trachomatis* pneumonia. To date, no erythromycin resistance has been reported among domestic strains, supporting its continued use as a first-line agent. In addition to its antibacterial activity, erythromycin has immunomodulatory properties that may confer additional benefit in this disease [18]. Bronchoscopy was performed in 14 patients in this cohort. It is not routinely indicated for the diagnosis of *C. trachomatis* pneumonia. In our practice, bronchoscopy was performed mainly for therapeutic and supportive purposes, including persistent wheezing or dyspnea requiring assessment of airway patency; atelectasis or consolidation requiring lavage to remove mucus plugs; and no improvement or disease progression despite standard treatment. When the pathogen was unclear, BALF was also used for pathogen detection. Therefore, bronchoscopy should be considered a supportive rather than a routine diagnostic procedure in infantile *C. trachomatis* pneumonia. Among the 11 children with CMV co-infection, 7 (63.6%) received ganciclovir, whereas the remaining 4 were considered to have asymptomatic infection, received no antiviral therapy, and recovered uneventfully. These observations suggest that CMV co-infection does not invariably require antiviral treatment. No deaths occurred, and the overall prognosis was favorable. Nevertheless, the need for respiratory support among patients with severe disease underscores the importance of close monitoring for subcostal retractions, tachypnea, and dyspnea, with prompt evaluation and transfer to intensive care when clinically indicated.

Limitations. Several limitations should be acknowledged. First, this was a single-center retrospective study with a relatively small sample size, and the enrollment period spanned nearly ten years (2016–2025), during which molecular diagnostics advanced rapidly. These factors may have limited statistical power and introduced temporal heterogeneity, although standard macrolide therapy and core management principles remained largely unchanged. Second, because the mothers of these infants delivered at other hospitals across China, it was impossible to ascertain whether they had undergone antenatal *C. trachomatis* screening or received antibiotic treatment, and the maternal infection rate could not be determined. Third, the follow-up period was limited, and long-term pulmonary function and respiratory sequelae were not systematically evaluated. Despite these limitations, this study has notable strengths, including a well-characterized cohort of infants with confirmed *C. trachomatis* pneumonia, comprehensive integration of clinical and imaging data, and detailed description of atypical HRCT patterns.

## 5. Conclusions

Infantile *C. trachomatis* pneumonia predominantly occurs within the first three months of life and is characterized by an afebrile or minimally febrile respiratory illness with cough, pulmonary crackles, and eosinophilia. Chest HRCT typically demonstrates bilateral patchy opacities and interstitial infiltrates; however, atypical manifestations, including mass-like opacities and miliary patterns, may also occur and should be differentiated from fungal and tuberculous infections. Tachypnea and retractions may serve as important early clinical indicators of severe disease and the potential need for respiratory support. Macrolide therapy is effective, and the overall prognosis is favorable. These findings support strengthening antenatal screening for *C. trachomatis* in obstetric practice and increasing pediatric awareness of atypical imaging manifestations. Infants presenting with signs of respiratory distress should be promptly assessed for disease severity and potential respiratory support requirements. Future multicenter prospective studies are needed to validate predictors of severe disease and characterize long-term outcomes.

## Figures and Tables

**Table 1:** Baseline characteristics of 30 infants with *C. trachomatis* pneumonia (n = 30).

Basic Characteristics	Value
Sex, male/female	16/14
Age at admission (days)	53.0 (43.5–62.0)
Age at onset (days)	34.0 (23.5–39.8)
Duration of illness before admission (days)	20.0 (10.3–29.0)
Length of hospital stay (days)	12.0 (9.0–15.8)
Full-term birth, n (%)	30 (100)
Vaginal delivery, n (%)	28 (93.3)
Cesarean section, n (%)	2 (6.7)
Birth weight (kg)	3.5 (3.1–3.7)
Maternal history of vaginitis, n (%)	15 (50.0)

Data are presented as median (interquartile range) for continuous variables and n (%) for categorical variables.

**Table 2:** Univariate logistic regression analysis of risk factors for severe disease.

Variable	OR	95% CI	*p* Value
Retractions	7.000	(1.296–56.189)	0.036
Feeding-associated regurgitation or choking	0.212	(0.037–0.988)	0.060
Wheezing	4.000	(0.891–20.697)	0.080
IgM	2.316	(0.969–6.836)	0.083

Abbreviations: OR, odds ratio; CI, confidence interval; IgM, immunoglobulin M. *p* values < 0.05 were considered statistically significant. Tachypnea showed complete separation (0% in non-severe vs. 92.9% in severe group); OR could not be reliably estimated.

## Data Availability

The data presented in this study are available on request from the corresponding author due to privacy and ethical restrictions. The data are not publicly available as they contain information that could compromise the privacy of research participants.

## Supplementary Materials

The following supporting information can be downloaded at: https://www.mdpi.com/article/10.3390/children13101416/s1, Table S1: Comparison of clinical characteristics between severe and non-severe groups.

## Abbreviations

The following abbreviations are used in this manuscript:

*C. trachomatis*	*Chlamydia trachomatis*
mNGS	Metagenomic next-generation sequencing
BALF	Bronchoalveolar lavage fluid
WBC	White blood cell count
Hb	Hemoglobin
CRP	C-reactive protein
PCT	Procalcitonin
ESR	Erythrocyte sedimentation rate
PaCO_2_	Arterial partial pressure of carbon dioxide
ALT	Alanine aminotransferase
AST	Aspartate aminotransferase
CK	Creatine kinase
CK-MB	Creatine kinase-MB
LDH	Lactate dehydrogenase
IgG	Immunoglobulin G
IgM	Immunoglobulin M
ORs	Odds ratios
CIs	Confidence intervals
CMV	Cytomegalovirus
HRCT	High-resolution computed tomography

## References

[1] Tu S., Li Y., Zhou Y., Zeng Z., Niu Y., Lin J., Chen J., Tang J., Liu P., Wen Y. (2026). Chlamydia beyond the genital tract: A major contributor to community-acquired pneumonia. Front. Cell. Infect. Microbiol..

[2] Tang C., Cai X., Niragire B., Louchez F.V., Levy-Zauberman Y.V., Subtil A., Wu Y. (2026). IFN-I exacerbates the inflammatory response of epithelial cells to *Chlamydia trachomatis* infection by enhancing TLR3 expression. mBio.

[3] González-Fernández M.D., Escarcega-Tame M.A., López-Hurtado M., Flores-Salazar V.R., Escobedo-Guerra M.R., Giono-Cerezo S., Guerra-Infante F.M. (2023). Identification of *Chlamydia trachomatis* genotypes in newborns with respiratory distress. An. Pediatr..

[4] Mohseni M., Sung S., Rausch-Phung E.A., Takov V. (2026). Chlamydia. StatPearls [Internet].

[5] Joly A., Rayment M. (2026). *Chlamydia trachomatis* and *Mycoplasma genitalium*. Clin. Dermatol..

[6] Liu Y., Xie H., Song Z., Huang M., Li M. (2026). Massive ascites and adnexal masses mimicking malignancy: A case report of *Chlamydia trachomatis* infection diagnosed by metagenomic next-generation sequencing. IDCases.

[7] Salari N., Olfat N., Ghasemi H., Larti M., Beiromvand M., Mohammadi M. (2025). The global prevalence of *Chlamydia trachomatis* genital infection in pregnant women: A meta-analysis. Arch. Gynecol. Obstet..

[8] Arteaga-Troncoso G., López-Hurtado M., Yescas-Buendía G., de Haro-Cruz M.J., Arteaga-Martínez I.A., Villagrana-Zesati J.R., Guerra-Infante F.M. (2024). Identifying the Impact of *Chlamydia trachomatis* Screening and Treatment on Mother-to-Child Transmission, and Respiratory Neonatal Outcomes in Mexico. Pathogens.

[9] Lanjouw E., Ouburg S., de Vries H.J., Stary A., Radcliffe K., Unemo M. (2016). 2015 European guideline on the management of *Chlamydia trachomatis* infections. Int. J. STD AIDS.

[10] Adachi K., Nielsen-Saines K., Klausner J.D. (2016). *Chlamydia trachomatis* Infection in Pregnancy: The Global Challenge of Preventing Adverse Pregnancy and Infant Outcomes in Sub-Saharan Africa and Asia. Biomed. Res. Int..

[11] Xu J., Yu L., Fu B., Zhao D., Liu F. (2018). Influence of different delivery modes on the clinical characteristics of *Chlamydia trachomatis* pneumonia. Eur. J. Pediatr..

[12] Li Y., Xiong L., Huang Y., Xia Y., Zhou H., Xu F., O’Sullivan M.V. (2015). The clinical characteristics and genotype distribution of *Chlamydia trachomatis* infection in infants less than six months of age hospitalized with pneumonia. Infect. Genet. Evol..

[13] Subspecialty Group of Respiratory, the Society of Pediatrics, Chinese Medical Association, Editorial Board, Chinese Journal of Pediatrics, China Medicine Education Association Committee on Pediatrics (2024). Guidelines for the management of community-acquired pneumonia in children (2024 revision). Zhonghua Er Ke Za Zhi.

[14] Souza E.L., Girão R.S., Simões J.M., Reis C.F., Galvão N.A., Andrade S.C., Werneck D.M., Araújo-Neto C.A., Souza L.S. (2012). *Chlamydia trachomatis*: A major agent of respiratory infections in infants from low-income families. J. Pediatr..

[15] Chen W., Dong H., Yang X. (2024). Risk factors of severe *Chlamydia trachomatis* pneumonia in children: A retrospective case-control study. J. Trop. Pediatr..

[16] Chiang Y.C., Shyur S.D., Huang L.H., Wen T.C., Yang H.C., Lin M.T., Liang P.H. (2005). *Chlamydia trachomatis* pneumonia: Experience in a medical center. Acta Paediatr. Taiwan.

[17] López-Hurtado M., Arteaga-Troncoso G., Sosa-González I.E., de Haro-Cruz M.d.J., Flores-Salazar V.R., Guerra-Infante F.M. (2016). Eosinophilia in Preterm Born Infants Infected with *Chlamydia trachomatis*. Fetal Pediatr. Pathol..

[18] Huang Y., Huang M., Ke L., Li S., Tao X., Zhu G. (2026). *Chlamydia trachomatis* Pneumonia in a 50-Day-Old Full-Term Male Infant Associated with Mucus Plugging of the Left Upper Lobe Bronchus and Left Upper Lobe Atelectasis. Am. J. Case Rep..

[19] Darville T. (2005). *Chlamydia trachomatis* infections in neonates and young children. Semin. Pediatr. Infect. Dis..

[20] Bekler C., Kultursay N., Ozacar T., Sayiner A., Yalaz M., Akisu M. (2012). Chlamydial infections in term and preterm neonates. Jpn. J. Infect. Dis..

