# Supplementary material for: Clinical Characteristics and Prognostic Analysis of Hospitalized Infants with Chlamydia trachomatis Pneumonia
Source: Children (Basel). 2026 Oct 15;13(10):1416. doi: 10.3390/children13101416 (PMC13703717; doi:10.3390/children13101416)
Supplement: Supplementary file 1 [file children-13-01416-s001.zip › children-4556427-supplementary.pdf]

Supplementary Table S1. Comparison of clinical characteristics between severe and non-severe groups.

Variables	Total (n = 30)	Non-severe (n = 16)	Severe (n = 14)	*p* value
**Demographics**				
Gender, n (%)				0.980
- Female	14 (46.7)	8 (50.0)	6 (42.9)	
- Male	16 (53.3)	8 (50.0)	8 (57.1)	
Age of onset (days)	34.0 (23.5–39.8)	31.5 (23.0–42.3)	35.5 (28.3–38.5)	0.829
Illness duration (days)	20.0 (10.3–29.0)	22.5 (11.8–30.0)	18.5 (10.0–20.0)	0.083
Length of hospital stay (days)	12.0 (9.0–15.8)	11.0 (9.0–16.5)	12.50 (9.3–13.8)	0.845
Birth weight (kg)	3.5 (3.1–3.7)	3.5 (3.2–3.8)	3.5 (2.9–3.6)	0.290
Feeding pattern, n (%)				0.722
- Breastfeeding	11 (36.7)	7 (43.8)	4 (28.6)	
- Formula feeding	2 (6.7)	1 (6.3)	1 (7.1)	
- Mixed feeding	17 (56.7)	8 (50.0)	9 (64.3)	
**Clinical manifestations, n (%)**				
Cough	29 (96.7)	15 (93.8)	14 (100.0)	1.000
Fever	13 (43.3)	7 (43.8)	6 (42.9)	1.000
Tachypnea	13 (43.3)	0 (0)	13 (92.9)	<0.001
Wheezing	12 (40.0)	4 (25.0)	8 (57.1)	0.156
Feeding-associated regurgitation or choking	12 (40.0)	9 (56.2)	3 (21.4)	0.117
Frothing at the mouth	6 (20.0)	4 (25.0)	2 (14.3)	0.784
Ocular discharge	5 (16.7)	3 (18.8)	2 (14.3)	1.000
Pertussis-like paroxysmal cough	3 (10.0)	2 (12.5)	1 (7.1)	1.000
Apnea	2 (6.7)	1 (6.2)	1 (7.1)	1.000
**Physical examination**				
Pulmonary crackles	26 (86.7)	14 (87.5)	12 (85.7)	1.000
wheezes	10 (33.3)	4 (25.0)	6 (42.9)	0.518
Retractions	9 (30.0)	2 (12.5)	7 (50.0)	0.046
Cyanosis	4 (13.3)	1 (6.2)	3 (21.4)	0.495
**Laboratory findings**				
WBC (× 10^9/L)	11.83 (9.43–14.29)	11.09 (9.32–13.25)	12.47 (11.42–15.02)	0.208
Eosinophils (× 10^9/L)	0.46 (0.21–0.84)	0.58 (0.24–0.82)	0.38 (0.11–0.54)	0.190
Hemoglobin (g/L)	103.5 (93.8–110.0)	105.5 (94.8–110.0)	99.5 (88.0–111.0)	0.482
CRP, n (%)				–
- Normal	30 (100)	16 (100)	14 (100)	
PCT, n (%)				0.967
- Normal	20 (66.7)	11 (68.8)	9 (64.3)	
- Elevated	2 (6.7)	1 (6.2)	1 (7.1)	
- Missing	8 (26.7)	4 (25.0)	4 (28.6)	
ESR (mm/h)	13.0 (6.0–27.0)	13.0 (6.5–14.0)	13.0 (5.0–27.0)	0.898
$PaCO_2$ (mmHg)	42.0 (36.0–49.0)	42.0 (36.0–48.0)	43.0 (36.5–49.0)	0.659
ALT (U/L)	30.75 (16.48–49.58)	30.75 (21.85–45.22)	33.70 (16.12–52.48)	0.959
AST (U/L)	48.80 (31.40–74.05)	41.20 (33.15–60.90)	51.90 (31.40–74.05)	0.751
CK (U/L)	93.5 (62.5–176.3)	71.5 (54.8–116.3)	121.5 (82.0–182.3)	0.123
CKMB (U/L)	21.5 (17.0–27.8)	21.5 (17.0–27.3)	21.5 (9.3–27.8)	0.323
LDH (U/L)	294.5 (254.0–355.5)	287.5 (253.3–349.5)	313.00 (256.8–355.5)	0.659
D-dimer, n (%)				0.781

- Normal	11 (36.7)	5 (31.2)	6 (42.9)	
- Elevated	19 (63.3)	11 (68.8)	8 (57.1)	
IgG (g/L)	11.20 (7.12–14.50)	10.60 (6.61–17.50)	11.25 (10.12–12.20)	0.669
IgM (g/L)	1.82 (1.28–2.61)	1.48 (1.24–1.99)	2.23 (1.42–3.36)	0.068
Co-infection, n (%)	13 (43.3)	7 (43.8)	6 (42.9)	1.000

Data are presented as median (interquartile range) for continuous variables and n (%) for categorical variables. — indicates not applicable or not calculated. Abbreviations: WBC, white blood cell; CRP, C-reactive protein; PCT, procalcitonin; ESR, erythrocyte sedimentation rate; $PaCO_2$, arterial partial pressure of carbon dioxide; ALT, alanine aminotransferase; AST, aspartate aminotransferase; CK, creatine kinase; CKMB, creatine kinase-MB; LDH, lactate dehydrogenase; IgG, immunoglobulin G; IgM, immunoglobulin M. Reference ranges: CRP < 10 mg/L; PCT < 0.5 ng/mL; D-dimer < 0.25 mg/L.
